# Effect of florfenicol on *nirS*-type denitrifying communities structure of water in an aquatic microcosm model

**DOI:** 10.3389/fvets.2023.1205394

**Published:** 2023-07-17

**Authors:** Tengyue Zhang, Jinju Peng, Yue Dai, Xingpeng Xie, Shuaishuai Luo, Yuexia Ding, Yi Ma

**Affiliations:** ^1^Department of Veterinary Medicine, College of Coastal Agricultural Sciences, Guangdong Ocean University, Zhanjiang, China; ^2^Maoming Branch, Guangdong Laboratory for Lingnan Modern Agriculture, Maoming, China

**Keywords:** florfenicol, nirS gene, denitrifying microbial community, water body microorganisms, aquatic microcosm model

## Abstract

Florfenicol is used worldwide for its low side effects and strong bactericidal effect. Florfenicol is physicochemically stable and can persist in natural water bodies and affect water denitrification. Indoor aquatic microcosm models were constructed and water samples were collected at different florfenicol concentrations (0.1, 1, 10, and 100 mg/L) on days 0, 7, 30, and 60 to extract the microbial genome DNA and determine the water properties. qPCR and amplicon sequencing were used to study the dynamic changes of *nirS* gene and *nirS*-type denitrifying communities structure, diversity and abundance, respectively. The results showed that higher florfenicol concentrations caused accumulation of nitrate and ammonium nitrogen in water. Florfenicol stress caused orders of magnitude changes in *nirS* gene abundance, showing a trend of increasing first and then decreasing. 100 mg/L florfenicol addition led to a sustained increase of *nirS* gene abundance in water bodies. The florfenicol addition altered denitrifying community structure and suppressed the richness and diversity index of denitrifying bacteria in water body. Over time, the richness and diversity index gradually recovered. *Proteobacteria* was always the dominant denitrifying phylum in water. The relative abundance of *Pseudomonas* and *beta proteobacterium* showed obvious positive correlation with *nirS* gene abundance and were the dominant genera under florfenicol stress. Our study provided a scientific basis for the rational use of florfenicol in aquaculture to maintain a healthy and stable microecological environment, and also provided a preliminary understanding of the response characteristics of water denitrifying microorganisms to florfenicol exposure.

## 1. Introduction

Aquaculture intensity requires the periodic use of antibiotics to treat and prevent bacterial disease outbreaks (1, 2). During farming, to ensure the proper growth of aquaculture animals, it also needs to be regularly replaced with an equal proportion of new water to treat the rapid accumulation of inorganic nitrogen compounds in the water (3, 4). According to the FAO 2020 report, the scale of worldwide aquaculture is increasing and is expected to reach 150 Mt by 2030 (5, 6). The rapid growth of intensive industrial aquaculture will produce large amounts of farm wastewater, causing natural water bodies to become eutrophic. Unused antibiotics can also eventually go back into the aquatic environment, making water bodies an important reservoir for antibiotics. The rapid growth of intensive industrial aquaculture will produce large amounts of farm wastewater, causing natural water bodies to become eutrophic. Unutilized antibiotics will also eventually enter the aquatic environment, causing water bodies to become important storage tanks for antibiotics (7). In addition, antibiotics can enter surface water, groundwater, and even possibly drinking water, which can pose a serious threat to human health and safety (8, 9).

Florfenicol shows low side effects and strong bactericidal activity and has been one of the antibiotics allowed to be used in aquaculture by the Food and Agriculture Organization of the United Nations (10). Since 2012, FLO has become the main antibiotic ingredient in fish feed premixes and is commonly recommended for fish bacterial diseases (11, 12), and the amount of florfenicol used in China is as high as 10,000 tons (13). However, researchers have shown that 45–60% of florfenicol cannot be absorbed by animals and is excreted in manure and urine (14). Furthermore, florfenicol is stable physicochemically and persists in aquatic systems under ambient temperature and pH conditions typical of natural waters (15). Different concentrations of florfenicol residues have been detected in coastal waters, sediments, and organisms (16). The residual concentration of florfenicol was as high as 11 mg/L in the waters around the Dalian Bay aquacultural farms in China (17). Six months after antibiotic treatment was stopped, the detected concentration of florfenicol was as high as 23.1 ng/L in salmon farms in Chile (18). Florfenicol was also detected in human urine and judged to be one of the antibiotics with a high health risk (19). Despite the high use of Florfenicol in farming all over the world, it is still a challenge to evaluate its negative environmental impacts, mainly on water bodies (20).

Denitrification, as the important nitrogen purification mechanism in lake waters (21), is the key part of the nitrogen cycle and the most economical approach to solving nitrate pollution in wastewater, which can purify water quality and alleviate water eutrophication (22). Denitrification is a series of enzymatic reactions mediated by microorganisms and involves four key enzymes: nitrate reductase, nitrite reductase, NO reductase and N_2_O reductase. Nitrite reductase, encoded by the *nirS* and *nirK* genes, is the rate-limiting enzyme for denitrification (23). The *nirS* and *nirK* genes have been used as functional markers for the presence of denitrification (24). The *nirS* gene is more broadly found among bacteria than *nirK* gene, and is more widely present in the physiological groups of bacteria (25). The *nirS* gene was used as a molecular marker to construct a clone library, which is an effective means to understand the composition and diversity of the denitrifying microbial community structure. Research has also reported varying degrees of adverse or inhibitory effects of antibiotics on denitrification (26, 27). Antibiotic residues can inhibit key enzyme activities of denitrifying microorganisms (27). However, it has also been reported that the negative effects of antibiotics decrease to some extent over time, and overall a gradual adaptation to the stress can be observed (26, 28). There is a lack of research regarding the effects of florfenicol on denitrifying microorganisms in the water body. In this study, an aquatic microcosm model was constructed to simulate an aquatic ecosystem with florfenicol added exogenously to explore the dynamic changes in water properties, *nirS* gene abundance, and the *nirS*-type denitrifying communities structure, abundance and diversity after different florfenicol concentrations were added to the aquatic environment. This study may provide theoretical data for the scientific use of florfenicol in aquaculture and the assessment of the ecological risk of florfenicol residues.

## 2. Materials and methods

### 2.1. Aquatic mesocosm experiment design and sampling

The mesocosm experiment was constructed to simulate the aquatic ecosystem. Surface water (1–10 cm) and surface sediments (1–10 cm) were collected from lakes at the Guangdong Ocean University (Zhanjiang, China), removing larger stones and other debris, and divided into transparent plastic boxes (50 × 40 × 30 cm). A plastic box contained 30 L of lake water and 10 cm of sediment. Keep at ambient temperature (25 ± 3°C) for 3 days until the aquatic mesocosm systems have stabilized. Florfenicol solution was added so that the concentration of florfenicol in the water column was 0, 0.1, 1, 10, and 100 mg/L. Water samples (numbered W0 to W4) were collected on days 0, 7, 30, and 60 (D0, D7, D30, and D60). Among them, the 0-day water samples (D0) were collected before the addition of florfenicol. The experiment had 5 groups with 3 replicates each. Three replicate samples from each group were mixed and immediately split into two parts for the determination of physicochemical parameters and DNA extraction.

### 2.2. Analysis of water sample properties

Several experiments were performed to assess the physiochemical characteristics of the samples. The pH and conductivity of the water samples were measured using a pH meter and conductivity meter, and nitrate nitrogen (colorimetric method) and ammonium nitrogen (indigo method) were estimated using an ultraviolet-visible spectrophotometer. Each sample was analyzed in triplicate.

### 2.3. DNA extraction and qPCR

According to the manufacturer's instructions, genomic DNA was extracted from water samples using the water DNA kit (Omega Bio-Tek, Norcross, GA, USA). The concentration and purity of the extracted DNA were assessed using a nanodrop UV-vis spectrophotometer. Three DNA of samples were extracted from each group for analysis. Based on previous work, the SYBR-primer method was employed with the CFX Connect Real-Time System instrument (BIO-RAD) to determine the abundance of the *nirS* gene. The denitrifying bacterial *nirS* gene was amplified using the primer pair CD3AF/R3CDR. The qPCR reaction system was: 10 μL SYBR, 1 μL DNA template, 1 μL upstream and downstream primers (10 mol/L) each, and 8 μL ddH_2_O. The qPCR reaction program was: 94°C, 5 min; 94°C for 30 s, 58°C for 30 s, 72°C for 30 s, 39 cycles.

### 2.4. Amplicon sequencing of *nirS* gene and bioinformatics processing

The DNA samples from each of the three replicates were combined into the mixed sample and sent to Jinweizhi (Suzhou, China) for Illumina MiSeq paired-end sequencing of the *nirS* gene amplicon regions. The *nirS* gene was amplified for sequencing analysis using the same primer pairs as qPCR. The high-throughput sequencing raw data was optimized using QIIME 1.9.1 by splicing overlapping regions at the ends of the sequences, removing sequences that were shorter than 200 bp, and removing chimeric sequences to produce valid data. An operational taxonomic unit (OTU) cluster analysis was carried out based on 97% similarity. The species taxonomic annotation was completed using the NCBI database. The 20 water samples were divided into different groups according to the sampling time and florfenicol addition concentration, and then OTU clustering analysis was performed. According to the OTU cluster analysis results, a Venn diagram was drawn. Based on OTU analysis results are obtained, using the method of random sampling sample sequences is flat, calculate Shannon, Chao1 alpha diversity index, community species abundance and diversity. Through the (UN) weighted unifrac analysis and comparison between samples whether there are significant differences in the microbial community. PCoA is display beta diversity visualization, PCoA is based on the distance between the matrix Brary - Curtis. Through the weighted clustering hierarchy and the group average method construct UPGMA (Unweighted pair group method with arithmetic mean) clustering tree. And diversity index was examined using QIIME 1.9.1 software and R language. The water properties and *nirS* gene abundance were used as independent variables, and denitrifying bacteria relative abundance was used as the response variable. By establishing a multiple linear regression model, the response variables were explained by the independent variables, and the correlation between them was explored.

### 2.5. Data statistic analyses

Excel 2016 was used to analyze the *nirS* gene abundance, R 3.3.1 was used to analyze alpha diversity, and the correlation between environmental factors and community structure was examined using CANOCO 5.0 software. The two-factor variance analysis was applied to test the effects of florfenicol concentration, time, and their interactions on water properties and *nirS* gene abundance. Statistical analyses were performed with IBM SPSS Statistics 24. All significant differences were at *P* ≤ 0.05.

## 3. Results

### 3.1. Effect of florfenicol on water properties

As shown in Table 1, florfenicol concentration and sampling time both affected clearly water properties. A higher effect on water properties was observed for sampling time than florfenicol addition concentrations. This trend was not broken by the lower florfenicol additions, but by the higher florfenicol additions. The pH of all water samples was alkaline and weakly alkaline, with a significant decrease in pH on day 7 and a gradual increase in water pH on days 30 and 60. The treatment groups with higher florfenicol additions showed a larger variation amplitude of pH. Higher concentrations of florfenicol caused an accumulation of nitrate nitrogen in the water compared to the control group. In addition, 100 mg/L florfenicol caused a marked accumulation of ammonium nitrogen and a marked rise in water conductivity at day 30. According to the statistical analysis results, concentrations of florfenicol and time significantly affected the water properties in an aquatic microcosm model (*P* < 0.05).

**Table 1:** Effect of florfenicol on water properties.

**Sample**	**pH**	**NO${\;}_{3}^{-}$-N (mg/L)**	**NH${\;}_{4}^{+}$-N (mg/L)**	**Conductivity (μS/cm)**
W0D0	8.45	4.02	0.27	493
W0D7	8.04	3.04	0.59	548
W0D30	8.50	1.66	0.23	622
W0D60	8.81	0.81	1.06	627
W1D0	8.55	3.94	0.26	506
W1D7	8.12	2.56	0.47	560
W1D30	8.48	1.49	0.32	614
W1D60	8.84	0.93	1.13	623
W2D0	8.69	3.36	0.58	517
W2D7	8.08	2.57	0.62	576
W2D30	8.56	1.91	0.46	634
W2D60	8.83	1.17	0.97	642
W3D0	8.58	3.61	0.51	493
W3D7	7.94	4.20	0.61	587
W3D30	8.73	8.83	0.60	660
W3D60	8.88	7.66	1.14	619
W4D0	8.57	3.08	0.40	516
W4D7	7.83	5.38	3.02	626
W4D30	8.87	12.48	7.27	841
W4D60	9.05	11.09	1.87	864

### 3.2. Effect of florfenicol on *nirS* gene abundance

**Figure 1:**
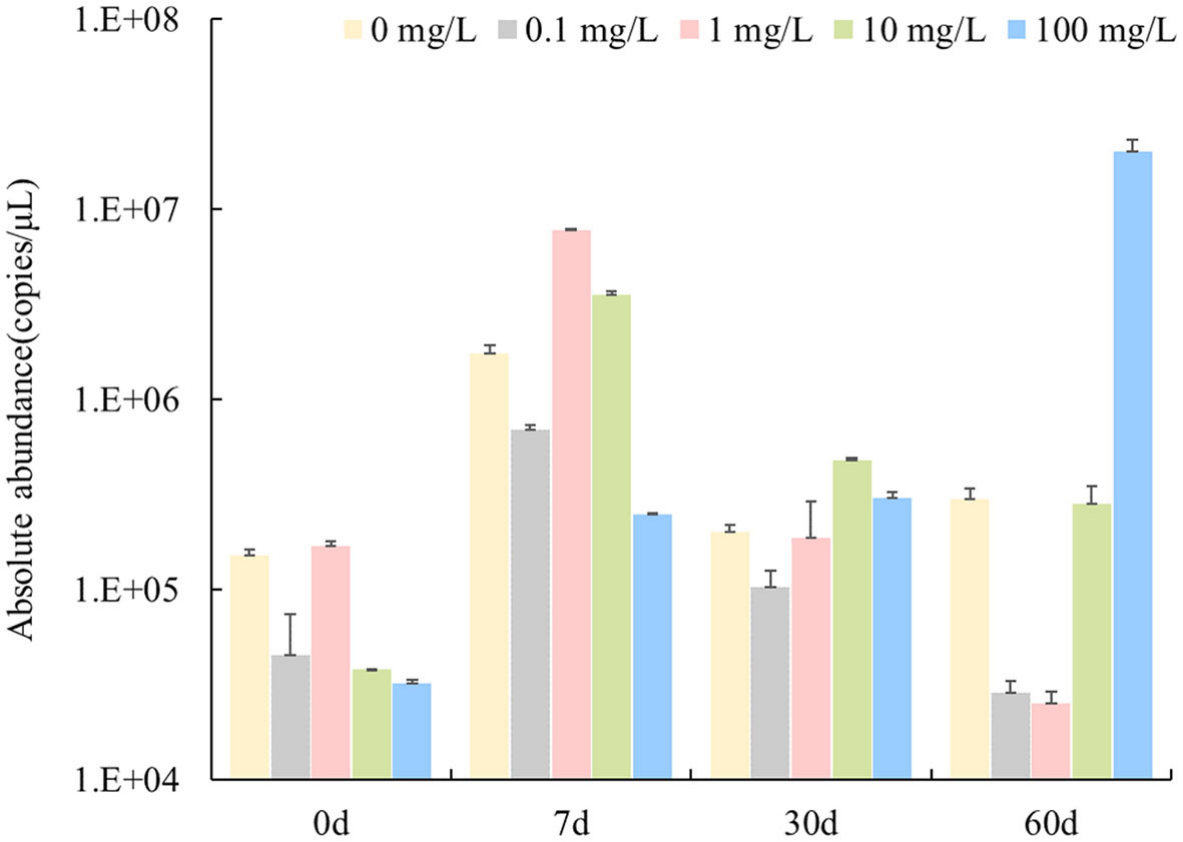
Effects of different concentrations of florfenicol on the abundance of *nirS* genes in an aquatic microcosm model.

As shown in Figure 1, the absolute abundance of *nirS* genes in the water bodies was 2.53 × 10^4^-2.02 × 10^7^ copies/μL. The *nirS* denitrifying community showed two response patterns to the florfenicol concentration in the water body when stressed by florfenicol. On Day 7, after the addition of florfenicol, the abundance of the *nirS* gene was 1–2 orders of magnitude higher. Moreover, the abundance of *nirS* gene increased more with increasing florfenicol addition concentration. On 30 and 60 days, the abundance of *nirS* gene decreased continuously over time, with a slower decrease in 10 mg/L florfenicol treatment groups. However, with 100 mg/L florfenicol, the abundance of *nirS* gene kept increasing over time until 60 days, when the abundance of *nirS* gene reached maximum value and increased by 3 orders of magnitude compared with no florfenicol addition. According to the statistical analysis results, concentrations of florfenicol and sampling time significantly affected the abundance of *nirS* genes in an aquatic microcosm model (*P* < 0.05).

### 3.3. Effect of florfenicol on *nirS*-type denitrifying communities structure

**Figure 2:**
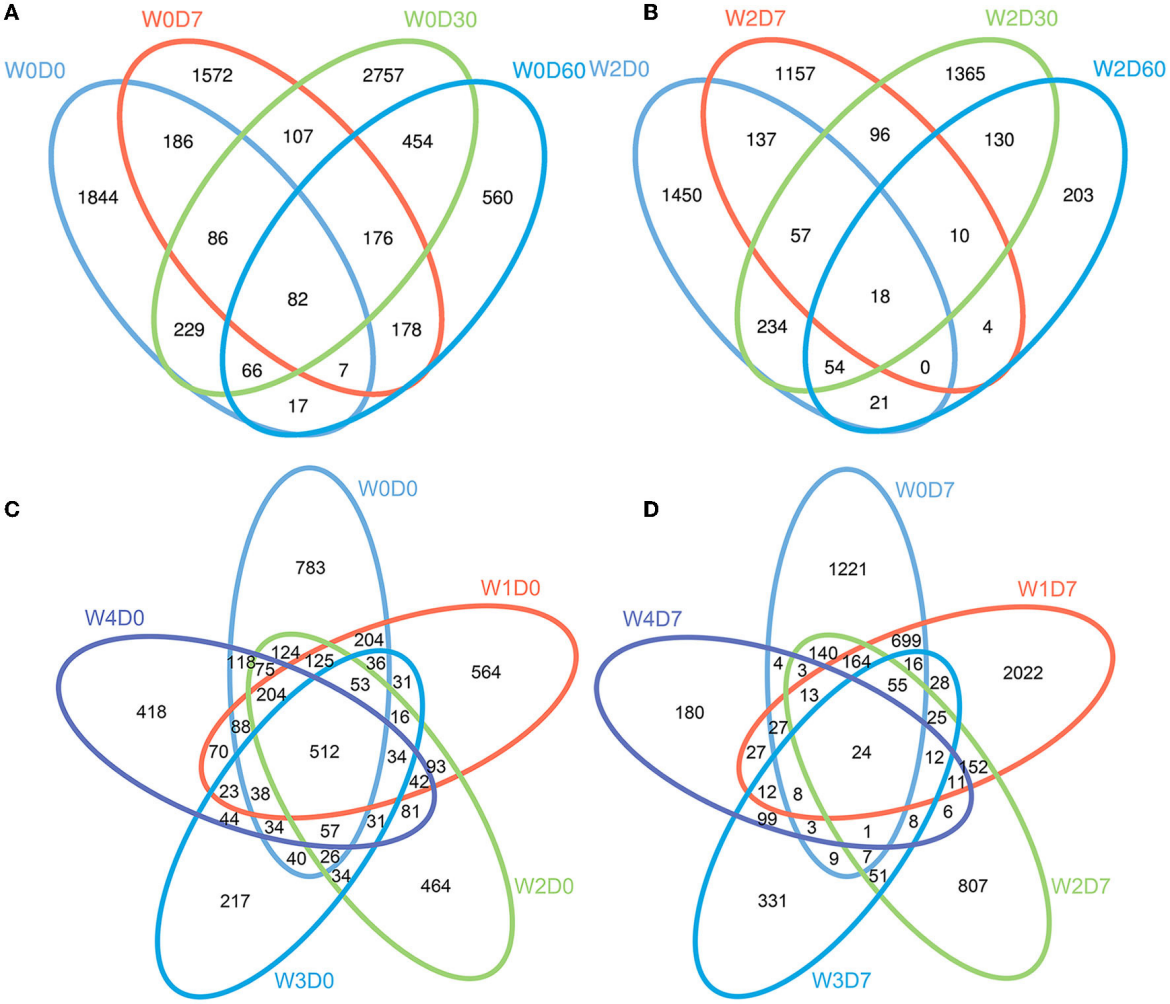
Effects of different concentrations of florfenicol on operational taxonomic units (OTUs) of bacteria (Venn diagram) in an aquatic microcosm model. **(A)** Effect of sampling time on the OUTs in group W0 samples. **(B)** Effect of sampling time on OUTs in group W2 samples. **(C)** OTUs differences between the 5 groups at 0 days. **(D)** Effects of different concentrations of florfenicol on OTUs at 7 days. Differently colored circles represent different samples, and the numbers represent the number of unique OTUs in each sample or the number of common OTUs in all samples.

After the quality optimization of the original data, the number of valid sequences obtained in water samples was 63,042–186,410. After OTU cluster analysis, the number of OTUs in each sample ranged from 285 to 2,607, with an average of 1,135 OTUs per sample, of which the W4D7 group had the fewest OTUs. The results of the cluster analysis showed that sampling time had an obvious influence on OTU composition in the water column without florfenicol. As shown in Figure 2, the 0-day samples had 512 common OTUs; the four samples without added florfenicol but collected at different times had 82 common OTUs. 1 mg/L florfenicol could markedly reduce the number of common OTUs. Florfenicol concentrations affect its degradation rate and produce different effects on microbial communities in the water body.

**Figure 3:**
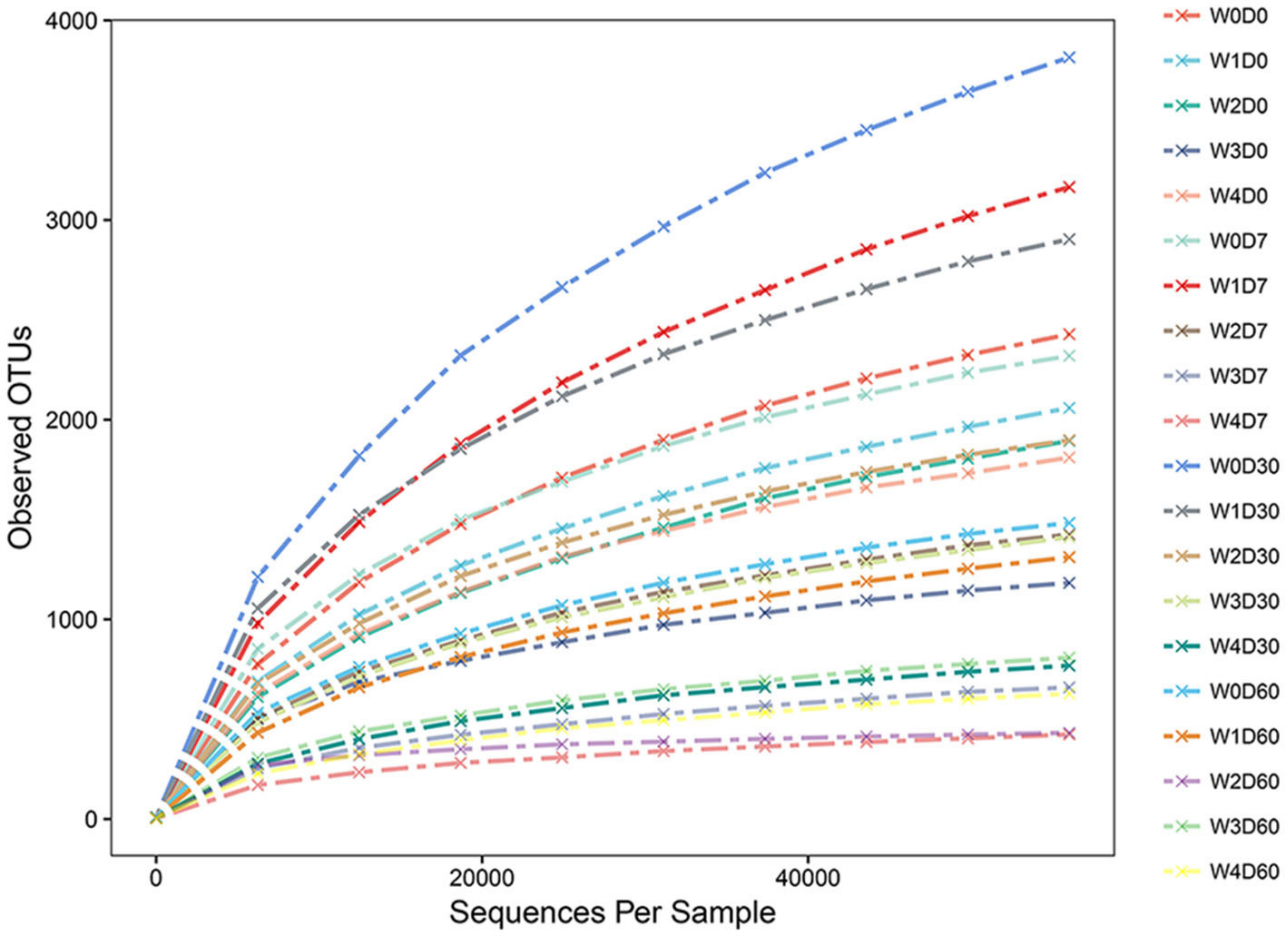
Effects of different concentrations of florfenicol on dilution curve of operational taxonomic units (OTUs) in an aquatic microcosm model.

The number of valid sequences and OTUs in each sample was obtained from the OTU analysis results. The random sampling method was used to extract a different number of sequences from each sample and calculate the number of OTUs after extraction. The dilution curve between OTU and the number of sample sequences was constructed by using the number of valid sequences extracted from the samples as the abscissa and the number of observed OTUs as the ordinate. As presented in Figure 3, with the increased number of extracted sequences, the number of detected OTUs increases and gradually tends to be flat, indicating that the sequencing is saturated, the sampling is essentially reasonable and the most abundant microbiome is included in the sequencing results. This provided a more realistic representation of the microbial community structure.

**Figure 4:**
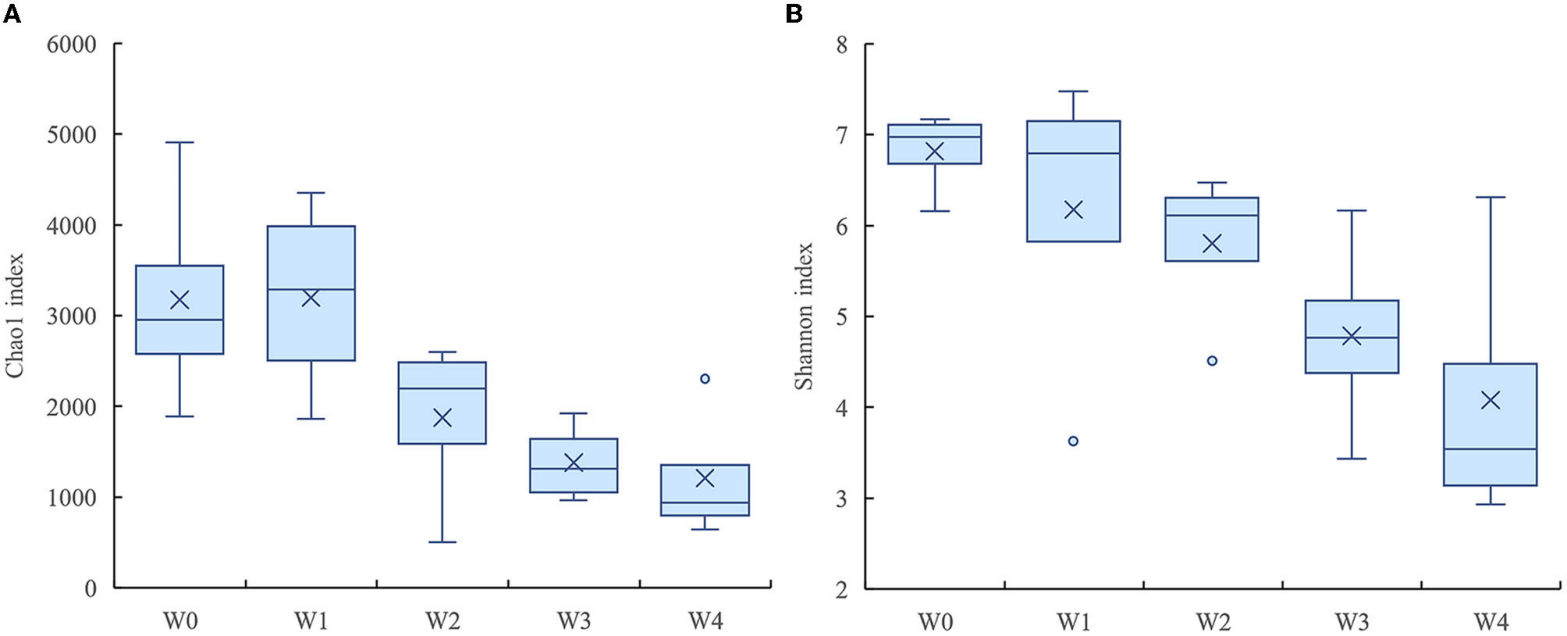
Effects of different concentrations of florfenicol on the Shannon index **(A)** and chao1 index **(B)** of denitrifying microorganisms in an aquatic microcosm model.

The denitrifying microbial community diversity indices mainly include the Shannon and Simpson indices, and the richness indices mainly include the Chao1 and ACE indices. Larger indices represent a greater total species number and higher community diversity. As shown in Table 2, denitrifying microbial community richness and diversity showed a decreasing trend in the water body as the concentration of florfenicol increased; with the change of time, the community richness and diversity were first suppressed and then gradually recovered. Its recovery rate was bounded by the florfenicol concentration. As shown in Figure 4, the Chao1 and Shannon indices of the denitrifying microbial community decreased with increased florfenicol concentrations. The most inhibition of denitrifying microbial community richness and diversity index was observed in the 100 mg/L florfenicol treatment group on day 7.

**Table 2:** Alpha diversity index of denitrifying microorganisms.

**Sample**	**OTUs**	**Chao1**	**Ace**	**Shannon**	**Simpson**
W0D0	1,693	3,098	3,265	6.86	0.97
W0D7	1,707	2809	3,043	7.09	0.96
W0D30	2,607	4,911	5,330	7.17	0.93
W0D60	1,052	1,885	2,038	6.16	0.96
W1D0	1,401	2,718	2,912	6.56	0.97
W1D7	2069	4,353	4,654	7.04	0.95
W1D30	2,025	3,860	4,051	7.48	0.97
W1D60	871	1,860	1,878	3.63	0.69
W2D0	1,244	2,596	2,783	6.25	0.95
W2D7	973	1,949	2,014	4.51	0.84
W2D30	1,347	2,450	2,561	6.47	0.96
W2D60	368	501	497	5.98	0.96
W3D0	856	1,546	1,631	6.17	0.95
W3D7	437	964	1,008	3.44	0.76
W3D30	954	1,923	2,013	4.85	0.88
W3D60	567	1,084	1,114	4.69	0.88
W4D0	1,291	2,306	2,386	6.31	0.95
W4D7	285	642	608	2.93	0.73
W4D30	528	1,032	1,085	3.87	0.80
W4D60	428	847	906	3.21	0.72

**Figure 5:**
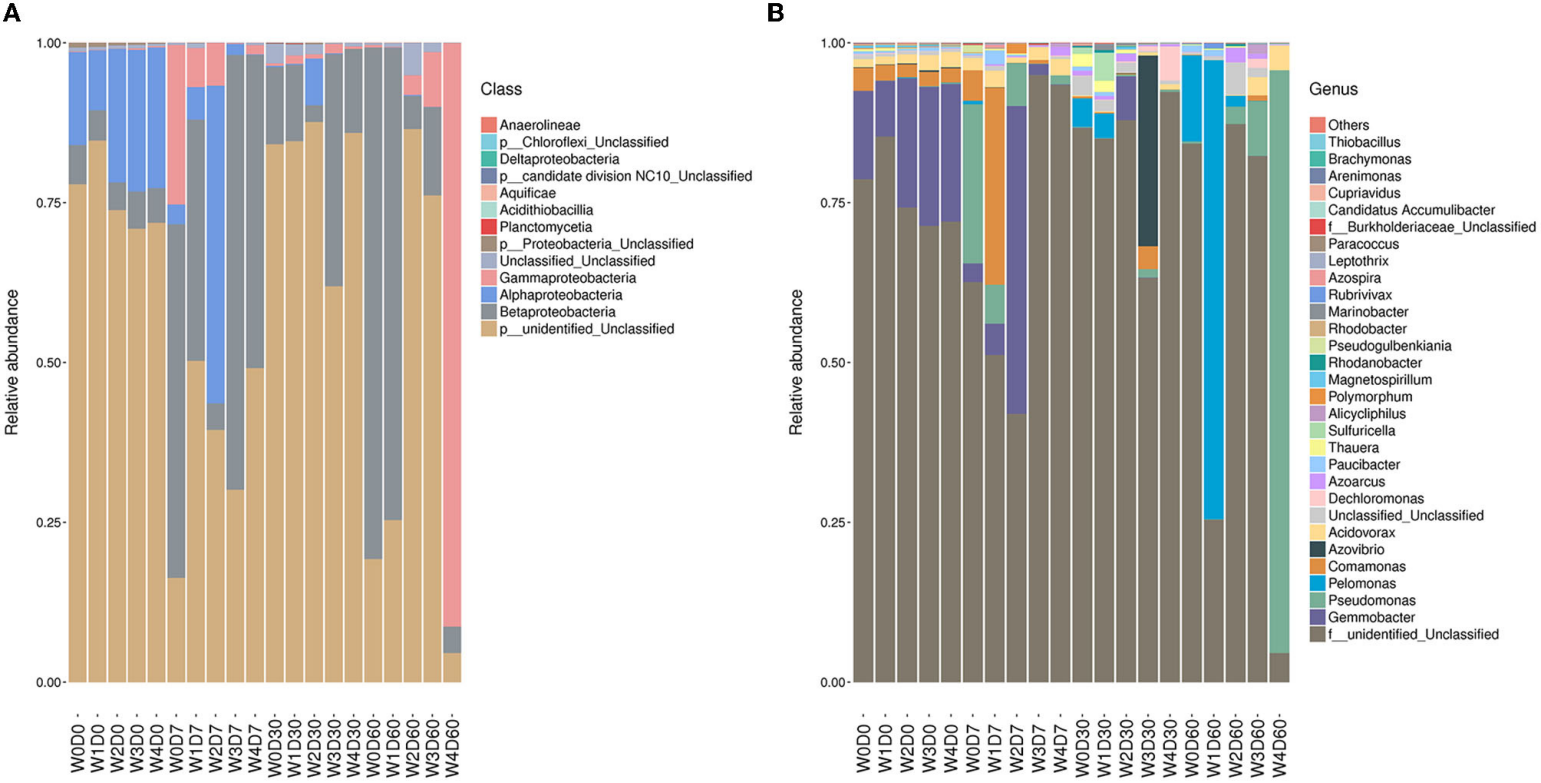
Effects of different concentrations of florfenicol on class-level **(A)** and genus-level **(B)** denitrifying microorganisms abundance in an aquatic microcosm model.

The denitrifying microbial community composition of each sample was statistical under different species classification levels, as shown in Figure 5. The identified species were classified into 7 phylum, 12 classes, 2 orders, 23 families, 52 genera, and 106 species. The relative abundance of each species in the denitrifying communities was calculated, with the relative abundance of unclassified species ranging from 4.57% to 87.83%. At the phylum level, the relative abundance of *Proteobacteria* ranged from 8.43% to 95.37% and mainly included *Betaproteobacteria, Alphaproteobacteria*, and *Gammaproteobacteria*, which were the absolute dominant bacteria. The relative abundance of *Betaproteobacteria* was the highest and the relative abundance of *Gammaproteobacteria* was the lowest, but the relative abundance of *Gammaproteobacteria* in the W4D60 treatment group was 91.22%. However, the relative abundance of *Gammaproteobacteria* was 91.22% in 60-day water sample with 100 mg/L florfenicol. At 7 days, the relative abundances of *Gemmobacter* and *Comamonas* were increased by lower florfenicol concentrations while higher florfenicol concentrations decreased the abundance of both. However, *Comamonas* was more sensitive to florfenicol than *Gemmobacter*, and 1 mg/L florfenicol reduced its abundance. The relative abundance of *Pseudomonas* and *Pelomonas* was < 0.2% on day 0, but they showed different responses to the florfenicol concentration on day 60. The relative abundance of *Pelomonas* was suppressed in groups with florfenicol added, except for 0.1 mg/L, where the relative abundance of *Pelomonas* reached 71.79%. The relative abundance of *Acidovorax* was not markedly changed at 7 days and markedly suppressed at 30 days, whereas higher florfenicol concentrations increased the relative abundance of *Acidovorax* at 60 days and returned to the 0-day level. In addition, the relative abundance of *Azovibrio* showed a steep increase to 29.84% in the 30-day water sample with 10 mg/L florfenicol. Higher florfenicol concentrations also caused enrichment of some unclassified species, for example, the relative abundance of OTU5, OTU13 and OTU14 could reach 41.83%, 25.77% and 37.55% respectively.

### 3.4. Beta diversity

**Figure 6:**
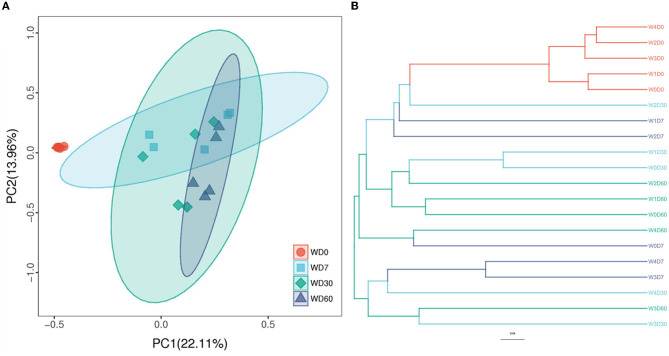
Principal Co-ordinates Analysis (PCoA) and unweighted pair group method with arithmetic mean (UPGMA) reveal the degree of similarity and difference of denitrifying microorganisms between different samples in an aquatic microcosm model. **(A)** The results of the PCoA analysis; **(B)** The results of the UPGMA analysis.

PCoA (Principal Co-ordinates Analysis) and UPGMA (Unweighted pair group method with arithmetic mean) clustering methods were used to express the similarity and differences degree between different samples. As shown in Figure 6A, the contribution of PC1 and PC2 principal coordinates to sample differences were 22.11% and 13.96% respectively in PCoA. The 0-day samples were completely separated from others samples collected at other times, indicating that sampling time had a greater effect on the degree of variation between samples. Samples with higher florfenicol concentrations added were closer together, where they had a higher similarity of denitrifying microbial communities. The similarity of denitrifying microbial communities in samples with lower florfenicol concentrations added was mainly influenced by time. Samples with similar denitrifying microbial community structure were clustered together in the clustering analysis. As shown in Figure 6B, 0-day samples were clustered together; 30- and 60-day samples with lower florfenicol concentrations were clustered with control (W0D30, W1D30, W0D60, W1D60 and W2D60); samples with higher florfenicol concentrations (W3D7, W4D7, W4D30W, W3D30, and W3D60) were clustered together.

### 3.5. Linkage among water properties, *nirS* gene abundance, and denitrifying communities

**Figure 7:**
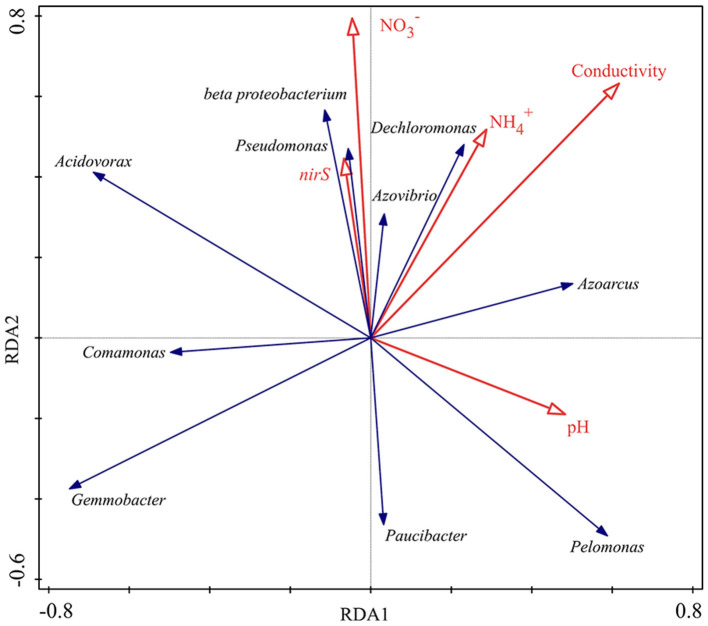
Correlation analysis between denitrifying communities and water properties, *nirS* gene abundance in an aquatic microcosm model.

The top 10 denitrifying bacteria with higher relative abundance in each sample were selected, and redundancy analysis (RAD) was used to investigate its correlation with water properties and *nirS* gene abundance. The results are shown in Figure 7, *beta proteobacterium* and *Pseudomonas* were strongly correlated with *nirS* gene abundance and showed a positive correlation, while *Gemmobacter, Pelomonas* and *Paucibacter* showed a clearly negative correction. *Pseudomonas, beta proteobacterium, Azovibrio* and *Dechloromonas* showed positive correlations with NO${\;}_{3}^{-}$ and NH${\;}_{4}^{+}$; while *Gemmobacter, Comamonas* and *Paucibacter* showed negative correlations. *Acidovorax* was sensitive to variations in pH and showed a significant negative correlation.

## 4. Discussion

Florfenicol inhibits the production of bacterial proteins by affecting the transpeptidase reaction of peptidyl transferase for bacterial inhibition. In aquaculture, florfenicol was fed in therapeutic doses together with feed. Bulk quantities of unabsorbed florfenicol persist in the water body until degradation, with the sediment carrying low florfenicol levels (29). The degradation products of florfenicol may still be biologically active in water-borne environments (15). The metabolic processes of microorganisms are also inevitably affected by the residual antibiotics in the water body. This study showed that 100 mg/L florfenicol caused the obvious change in water properties, massive accumulation of nitrate and ammonium nitrogen, increase in conductivity, and a dramatic change in water pH. The denitrifying enzyme activities are closely linked to substrate concentrations (30). Dynamic changes in substrate concentrations lead to variation in denitrifying enzyme activities, and the sensitivities of denitrifying enzymes activities to environmental conditions are different with different substrate concentrations (31). It has been shown that nitrate reduction might be the rate-limiting step in the denitrification process (32), so it was confirmed that high florfenicol concentrations hindered the water denitrification process and inhibited nitrate reductase activity in our study. Many studies have also reported the inhibitory effect of antibiotics on denitrification. For example, sulfamethoxazole, ciprofloxacin, amoxicillin and aureomycin showed various inhibition on the denitrification performance of microorganisms in short-term acute stress experiments and led to a reduction in nitrate removal (32). Chen et al. (31) reported that norfloxacin inhibited nitrate reduction and nitrate reductase activity, and enhanced nitrite reductase activity to some extent.

The inhibition of denitrification of antibiotics may be achieved by affecting bacterial growth, gene expression, denitrifying enzyme activity, and electron transport system activity (31). The dynamics of *nirS* genes, which encode key enzymes for denitrification, were quantified by qPCR. The results showed that florfenicol addition stimulated different increases of *nirS* gene abundance in water body. The abundance of *nirS* genes showed two response patterns to different florfenicol concentrations in aquatic microcosm models. Apart from 100 mg/L florfenicol, *nirS* genes abundance in water tended to increase and then decrease over time; it showed orders of magnitude increase at day 7; and the higher florfenicol concentration was used, the more obvious gene abundance increase compared with the control. While 100 mg/L florfenicol addition led to a sustained increase of *nirS* gene abundance throughout all experimental phases. It has been shown that florfenicol slightly promoted the nitrite reductase-coding gene in a short time (25, 32). As shown by Xie and Wang (33, 34), florfenicol promoted the *nirK* gene in sediment. However, the richness and diversity of denitrifying microorganisms showed an opposite trend to *nirS* genes in our experiment. Florfenicol inhibited the richness and diversity of denitrifying microorganisms while stimulating the abundance of *nirS* genes to increase, but the inhibition recovered slowly with time. Higher florfenicol concentrations resulted in greater inhibition of richness and diversity indices and a slower recovery process. This is in line with our previous findings regarding the effects of florfenicol on the richness and diversity of bacteria in water bodies (35). The inhibition of denitrifying microorganisms caused by florfenicol may lead to reduced denitrification capacity in water bodies. An et al. reported that sulfamethoxazole inhibited the denitrifying process by effectively suppressing the expression of denitrifying genes, rather than a reduce in total denitrifying microorganism abundance (36). This differs from our experimental results.

Effects of antibiotics on the denitrification process are mainly attributed to influences of denitrifying microorganisms. Florfenicol significantly affected the bacterial community structure and even led to a complete reversal of the microbial community (35). In this study florfenicol also had a significant effect on the water denitrifying microbial community structure, with higher and lower concentrations of florfenicol showed two developmental trends in the water denitrifying microbial community evolution over time. *Pseudomonas* is the dominant bacterium under florfenicol stress and considered as a potential host bacterium for florfenicol resistance genes. In this study, we found that *Pseudomonas* was also an important denitrifying bacterium under high florfenicol concentrations. This may be due to its ability to carry both the florfenicol resistance gene and the denitrification function gene, which could become the superior bacteria under florfenicol stress.

The studies have been reported about the effects and mechanisms of types, addition times and concentrations of antibiotics on denitrification in different environmental media such as sediment, soil, groundwater and wastewater (37). Different conclusions have also been drawn from the effects of antibiotics on denitrification, including promotion, no significant effect and inhibition. However, there are limited studies on the effects of florfenicol on denitrification, and its effects on water denitrification have not been reported. Our study filled this gap and provided preliminary understanding of the response characteristics of water denitrifying microorganisms to florfenicol exposure. Our study provided a preliminary understanding of the response characteristics of water denitrifying microorganisms to florfenicol exposure.

## 5. Conclusion

In this study, florfenicol additions changed the water properties and disrupted the original community structure. High florfenicol concentrations caused a massive accumulation of nitrate and ammonium nitrogen. *nirS* gene abundance showed orders of magnitude changes. Two trends in *nirS* gene abundance were observed, increased then decreased and increased continuously, depending on florfenicol concentrations. Florfenicol addition reduced the diversity and richness of *nirS*-type denitrifying microbial communities, which gradually recovered over time.

## Data availability statement

The datasets presented in this study can be found in online repositories. The names of the repository/repositories and accession number(s) can be found below: https://www.ncbi.nlm.nih.gov/, PRJNA943538.

## Author contributions

Funding acquisition and supervision: YM. Investigation and writing-original draft: TZ. Methodology: TZ and JP. Project administration and data curation: YDi and YDa. Software and visualization: SL and XX. Writing-review and editing: JP and YM. All authors have read and agreed to the published version of the manuscript.
